# Internet use and physical activity of older adults during the COVID-19 pandemic: a cross-sectional study in a northern Japanese City

**DOI:** 10.1186/s12877-022-03360-5

**Published:** 2022-08-19

**Authors:** Sachiko Sasaki, Akinori Sato, Yoshie Tanabe, Shinji Matsuoka, Atsuhiro Adachi, Toshiya Kayano, Hiroshi Yamazaki, Yuichi Matsuno, Ann Nakano, Toshihiro Watanabe

**Affiliations:** 1 Department of Physical Therapy, Faculty of Human Sciences, Hokkaido Bunkyo University, 5-196-1 Kogane-chuo, Eniwa, 061-1449 Japan; 2 Department of Health and Welfare, Eniwa City Hall, 1 Kyo-machi, Eniwa, 061-1444 Japan; 3 Department of Health and Nutrition, Faculty of Human Sciences, Hokkaido Bunkyo University, 5-196-1 Kogane-chuo, Eniwa, 061-1449 Japan

**Keywords:** COVID-19, Internet use; information and communication technology, Motor activity, Physical activity, Older adults, Community-dwelling

## Abstract

**Background:**

Little is known of whether Internet use is associated with physical activity among socially isolated older adults during the coronavirus disease 2019 (COVID-19) pandemic. This study investigated the association between Internet use and physical activity, and whether this association differs depending on social isolation among community-dwelling Japanese older adults.

**Methods:**

A cross-sectional study was conducted with 1048 community-dwelling residents aged 65–90 years. Data were obtained using a self-reported questionnaire in August 2020. Physical activity was assessed using the International Physical Activity Questionnaire-Short Form. Multivariable logistic regression analyses were used to calculate the odds ratios (ORs) and 95% confidence intervals (CIs) for the associations between Internet use and moderate-to-vigorous physical activity (MVPA).

**Results:**

Internet use showed a significant association with MVPA (OR = 1.42, 95% CI: 1.06–1.90) after adjusting for age, sex, self-reported socioeconomic status, and other health-related characteristics. When the results were stratified by social participation and living status, Internet use was associated with a significantly higher likelihood of MVPA among participants with no social participation (OR = 1.81, 95% CI: 1.03–3.17) and living with family (OR = 1.40, 95% CI: 1.02–1.93).

**Conclusion:**

Internet use was associated with sufficient physical activity, and this association may differ depending on the social isolation among community-dwelling older adults in Japan.

## Background

Since the beginning of 2020, there have been concerns that the amount of physical activity older adults engage in is decreasing due to the lockdowns and physical and social distancing measures introduced to reduce the transmission of coronavirus disease 2019 (COVID-19). Recent studies have shown that, in Japan and worldwide, physical activity of all intensity levels (vigorous, moderate, walking, and overall) has decreased and daily sitting time has increased for older people compared to pre-pandemic period [1–3]. In our recent study, physical activity among the older adults in northern Japan decreased by 5–10% compared to its level at pre-pandemic period even when social restrictions were partially lifted [4]. Physical activity is an important determinant of health and daily physical activity prevents many diseases such as cardiovascular disease [5], type 2 diabetes mellitus [6], and reduces mortality [7–9]. Thus, the reduction in physical activity due to the pandemic could have a considerable impact on the long-term health of the elderly. Consequently, several prospective studies have revealed that understanding the determinants of physical activity among the elderly is critical to the promotion of physical activity and the development of public health interventions. Moreover, several cross-sectional studies indicate that older people with lower social participation and living alone, which are factors representing the social limitations of older people, are more likely to be less physically active during the pandemic [10, 11]. Social limitation in older people is an important contributing factor for promoting physical activity [12], and it has an adverse effects on health such as decreasing mortality [13] and coronary heart disease [14] and improving physical [15] and cognitive function [16]. Therefore, more interventions are needed to maintain and increase participation in physical activity for those who are socially isolated during prolonged pandemics.

Although vaccination has minimized, to some extent, the lifestyle restrictions the older adults due to COVID-19 [17], the epidemic is not yet fully under control, not only in Japan but worldwide [18], and it is necessary to note that once the pandemic resurfaces, the older adults, who are at high risk of serious illness due to infection, will be forced to limit their outings and social activities. Therefore, there has been increasing recognition of the importance of Internet use as a social determinant of health [19] and a countermeasure for the decline in physical activity in older adults. Use of the Internet, including social media, and video tools may help older adults obtain information on how to maintain an appropriate social distance and engage in moderate physical activity [20]. A recent cross-sectional study involving community-dwelling Japanese older adults showed that the use of information and communications technology (ICT), including the Internet, was associated with voluntary exercise during the pandemic [21]. However, to date, no research has examined whether Internet use is associated with increased physical activity, especially among socially isolated older adults during the pandemic. Therefore, the present study investigated the association between Internet use and physical activity in community-dwelling Japanese older adults. Moreover, we examined whether this association differed based on social isolation.

## Methods

### Study design and population

A cross-sectional, community-based study was conducted in August 2020 in Eniwa, Hokkaido Prefecture, Japan [4]. With reference to a previous study on information and communication technology among Japanese older adults [21], we assumed that a 10% difference in physical activity by internet use could also be used in the current study; thus, the required sample size when α = 0.05 and β = 0.10 was 400 individuals who participate in internet use. This study involved community-dwelling men and women aged 65–90 years, except for those living in nursing homes or eligible for elderly care. A stratified sampling method was used. Respondents were randomly selected based on a list maintained by the Eniwa City Hall in terms of gender, age (65–74, 75–90), region of residence (one of four regions), and social participation prior to the COVID-19 epidemic. We considered 2008 older adults as eligible for inclusion and mailed a self-administered questionnaire inquiring about their age, sex, self-reported health status, smoking status, educational background, depression status, family members, socioeconomic status, and social participation. A total of 1493 participants (624 men and 869 women) completed the questionnaire (response rate: 74.4%). Of these, we excluded 195 because of missing physical activity data and 250 because of missing other types of data. Finally, a total of 1048 participants (491 men and 557 women) were included in the analysis.

### Assessment of internet use

Internet use was assessed using the question, “Have you used the Internet or e-mail in the past year? Please include the frequency of use.” The following four response options were provided: “No, I do not use the Internet,” “Yes, I use the Internet a few times a month,” “Yes, I use the Internet two or three times a week,” and “Yes, I use the Internet almost every day.” We considered that the latter three answers indicate ICT users. The following five answers helped evaluate when the participants started using the Internet and e-mail: “more than 10 years ago,” “more than 5 years ago,” “3 to 4 years ago,” “1 to 2 years ago,” and “less than 1 year ago.” Furthermore, participants were asked about the purpose of their Internet use through the following response options: “gathering/searching information on health and medical care,” “gathering and searching for various types of information” (excluding health and medical care), “communicating,” “purchasing products and services,” “conducting bank transactions,” “conducting stock and securities transactions,” “using social media service,” and “other.”

### Assessment of physical activity

Physical activity was assessed using the International Physical Activity Questionnaire-Short Form (IPAQ-SF) [22], The Japanese version of the IPAQ-SF was prepared by translating the original text into Japanese and then back-translating it into English, and it has shown adequate validity to measure physical activity and sedentary behavior in older adults in Japan (*r* = 0.42–0.53) [23]. Participants were asked to report the frequency and duration of any walking and moderate-to-vigorous physical activity (MVPA) performed for more than 10 continuous minutes during a typical seven-day period. Standardized MET values were assigned for walking (3.3 METs), moderate-intensity activity (4.0 METs), and vigorous-intensity activity (8.0 METs), and the total metabolic equivalents (METs) in minutes per week were calculated in accordance with the official IPAQ-SF guidelines. Additionally, the obtained data were classified into three categories based on the guidelines: low, moderate, and high. We also categorized the physical activity level into two categories—low physical activity and MVPA — [24] because current activity guidelines recommend MVPA as a key determinant of health in older adults [3]. Additionally, the total sitting time per week was measured with the question, “How much time did you usually spend sitting during a weekday?”

### Assessment of other factors

A self-administered questionnaire was used to collect data, and the following variables were considered as covariates: age, sex, self-reported health status, smoking status, educational background, economic status, depression status, family structure, and social participation. Self-reported health status was categorized as “good” or “poor.” Smoking status was categorized “yes” or “no.” Education level was categorized as “high school or less” or “more than high school.” Economic status was assessed using a subjective measurement through the question, “How do you feel about your current economic situation?” The five response options were “excellent,” “good,” “normal,” “poor,” and “very poor.” We classified excellent, good, and normal as “normal to good,” and poor and very poor as “poor” [25]. Depression status was assessed using the Geriatric Depression Scale-Short Form, and the scores ranged from 0 to 15 points, with higher scores representing a more depressed state, and the internal consistency was assessed [26, 27]; those with > 5 points overall were considered as being depressed. Social participation was defined as participation at least once a month in any of the following activities: volunteer groups, sports groups or clubs, hobby groups, senior citizen clubs, neighborhood associations/community groups, study or cultural clubs, health promotion activities, and income-generating work because previous studies among Asian older populations showed that those who participated in any of these social activities at least once a month and continuously participated in social activities had better mental and physical health [28, 29]. Family structure was categorized as “living alone” or “not living alone.”

### Statistical analysis

The data are presented as the mean ± standard deviation and the percentage (number) of participants in that category. We examined the association between Internet use and MVPA using a logistic regression model. Further, we calculated the odds ratios and 95% confidence interval (CIs) of MVPA for Internet use using the following models, selected based on previous studies [3, 4, 21, 30, 31]. Model 1 was adjusted for age (year as a continuous variable) and sex. Model 2 was adjusted for model 1 variables plus the self-reported health status (good or poor), current smoking status (yes or no), education level (high school or less or more than high school), economic status (good or poor), and depression status (more or less than 5 points). Model 3 was adjusted for model 2 variables plus social participation (yes or no) and living alone (yes or no). Next, to assess whether the association between Internet use and physical activity was modified by social isolation, we performed stratified analyses based on social participation and living alone. All analyses were conducted using JMP Pro software version 16.0.0 for Macintosh (SAS Institute, Cary, NC, USA). Statistical significance was defined as a two-tailed *p*-value of < 0.05.

## Results

In the overall sample, the mean age ± standard deviation was 74.4 ± 6.4 years, and 43.7% (52.6% men and 35.9% women) of all participants were Internet users. Most Internet users had used the Internet for more than a year, and 65.1% had used it for more than 10 years. The most common purpose of Internet use was to gather information, including medical and health information, accounting for 62.2% of all Internet users.

Table 1 shows the characteristics of the 1048 (491 men and 557 women) participants by Internet use. Participants who used the Internet were more likely to have good health status, more education, more social participation, and less depression and were less likely to live alone than non-Internet users. In addition, Internet users had a longer mean of vigorous, moderate, and total physical activity (expressed as METs in minutes per week) than non-Internet users did.

**Table 1:** Association of older adults’ demographic characteristics and Internet use

	Non-Internet Users	Internet Users	*p-value for difference*
	(*n =* 590)	(*n =* 458)	
Age (years)	76.8 ± 6.3	71.3 ± 5.0	*< 0.001*
Women (%)	357 (60.5)	200 (43.7)	*< 0.001*
Self-reported health (%)			*< 0.001*
Good	463 (78.5)	404 (88.2)	
Poor	127 (21.5)	54 (11.8)	
Smoking status (%)			0.78
Non-smoker	532 (90.2)	410 (89.5)	
Current smoker	58 (9.8)	48 (10.5)	
Educational background (%)			*< 0.001*
< High school	503 (85.3)	288 (62.9)	
> High school	87 (14.7)	170 (37.1)	
Economic status (%)			0.45
Normal to good	487 (82.5)	386 (84.3)	
Poor	103 (17.5)	72 (15.7)	
Depression (%)			*< 0.001*
No	285 (48.3)	287 (62.7)	
Yes	305 (51.7)	171 (37.3)	
Social participation (%)			*< 0.001*
No	217 (36.8)	86 (18.8)	
Yes	373 (63.2)	372 (81.2)	
Living alone (%)			0.011
No	487 (82.5)	404 (88.2)	
Yes	103 (17.5)	54 (11.8)	
METs of physical activity (METs･minutes/week)			
Vigorous intensity	991.8 ± 2073.9	1324.3 ± 2373.6	0.016
Moderate intensity	607.5 ± 1075.5	993.3 ± 1231.7	*< 0.001*
Walking	687.9 ± 964.3	797.4 ± 948.0	0.066
Total physical activity	2287.2 ± 3343.7	3115.0 ± 3572.4	*< 0.001*
Sitting time (minutes/day)	278.8 ± 199.5	275.5 ± 194.2	0.79

Variables are presented as the mean ± standard deviation or as number (%) of participants in that category. *Student’s t-test, chi-squared test was used to compare participant characteristics between internet use*

As Table 2 shows, the odds ratio for MVPA by Internet use among all participants was 1.49 (95% CI: 1.12–1.99, *p* = 0.007) after adjusting for possible confounding factors, including age, sex, self-reported health, smoking, educational background, subjective economic status, and depression status (model 2). The odds ratio did not change significantly and was 1.42 (95% CI: 1.06–1.90, *p* = 0.020) even after further adjusting for social participation and living alone (model 3). The goodness of fit for the models by the likelihood ratio test were significant (*P* <  0.05) for each model.

**Table 2:** Prevalence and odds ratio for MVPA for 1048 older adult participants according to Internet use

	Non-Internet Users	Internet Users	*P*-value
	(*n =* 590)	(*n =* 458)	
MVPA, n	274	282	
Crude odds ratio (95% CI)	1.00 (Reference)	1.85 (1.44, 2.37)	< 0.001
Adjusted odds ratio (95%CI), model 1	1.00 (Reference)	1.57 (1.19, 2.07)	0.001
Adjusted odds ratio (95%CI), model 2	1.00 (Reference)	1.49 (1.12, 1.99)	0.007
Adjusted odds ratio (95%CI), model 3	1.00 (Reference)	1.42 (1.06, 1.90)	0.020

A logistic regression model was used to calculate the odds ratios. Model 1 was adjusted for age and sex. Model 2 was adjusted for the same covariates used in model 1 plus self-reported health, smoking, educational background, subjective economic status, and depression status. Model 3 was adjusted for the same covariates used in model 2 plus social participation and living alone. MVPA: moderate-to-vigorous physical activity; CI: confidence interval

Table 3 presents the results of further analyses stratified by social participation. For participants with no social activities, the odds ratio of MVPA associated with Internet use was 1.80 (95% CI: 1.03–3.16, *p* = 0.041) after adjusting for possible confounding factors, including age, sex, self-reported health, smoking, educational background, subjective economic status, and depression status (model 2). The odds ratio did not change and was 1.81 (95% CI: 1.03–3.17, *p* = 0.040) even after further adjusting for living alone (model 3). By contrast, Internet use was not associated with MVPA among participants with social participation (odds ratio = 1.28, 95% CI: 0.90–1.80, *p* = 0.17). The goodness of fit for the models by the likelihood ratio test were significant (*P* <  0.05) for each model.

**Table 3:** Prevalence and odds ratios for MVPA in 1048 older adult participants according to Internet use by social participation

	Non-Internet Users	Internet Users	*P*-value
	(*n =* 590)	(*n =* 458)	
Social participation (no)			
MVPA, n	83	49	
Crude odds ratio (95% CI)	1.00 (Reference)	2.14 (1.29, 3.57)	0.003
Adjusted odds ratio (95%CI), model 1	1.00 (Reference)	1.91 (1.11, 3.30)	0.020
Adjusted odds ratio (95%CI), model 2	1.00 (Reference)	1.80 (1.03, 3.16)	0.041
Adjusted odds ratio (95%CI), model 3	1.00 (Reference)	1.81 (1.03, 3.17)	0.040
Social participation (yes)			
MVPA, n	191	233	
Crude odds ratio (95% CI)	1.00 (Reference)	1.60 (1.19, 2.14)	0.002
Adjusted odds ratio (95%CI), model 1	1.00 (Reference)	1.29 (0.93, 1.81)	0.13
Adjusted odds ratio (95%CI), model 2	1.00 (Reference)	1.27 (0.90, 1.80)	0.21
Adjusted odds ratio (95%CI), model 3	1.00 (Reference)	1.28 (0.90, 1.80)	0.17

A logistic regression model was used to calculate the odds ratios. Model 1 was adjusted for age and sex. Model 2 was adjusted for the same covariates used in model 1 plus self-reported health, smoking, educational background, subjective economic status, and depression status. Model 3 was adjusted for the same covariates used in model 2 plus living alone. MVPA: moderate-to-vigorous physical activity; CI: confidence interval

Next, Table 4 presents the results of analyses stratified by living alone. For participants not living alone, the odds ratio of MVPA associated with Internet use was 1.48 (95% CI: 1.08–2.03, *p* = 0.014) after adjusting for possible confounding factors, including age, sex, self-reported health, smoking, educational background, subjective economic status, and depression status (model 2). The odds ratio did not change much at 1.40 (95% CI: 1.02–1.93, *p* = 0.037) even after further adjusting for social participation (model 3). By contrast, Internet use was not associated with MVPA among participants living alone (odds ratio = 1.60, 95% CI: 0.72–3.55, *p* = 0.25). The goodness of fit for the models by the likelihood ratio test were significant (*P* <  0.05) for each model.

**Table 4:** Prevalence and odds ratios for MVPA in 1048 older adult participants according to Internet use by living alone

	Non-Internet Users	Internet Users	*P*-value
	(*n =* 590)	(*n =* 458)	
Living alone (no)			
MVPA, n	231	251	
Crude odds ratio (95% CI)	1.00 (Reference)	1.82 (1.39–2.38)	< 0.001
Adjusted odds ratio (95%CI), model 1	1.00 (Reference)	1.57 (1.16, 2.11)	0.003
Adjusted odds ratio (95%CI), model 2	1.00 (Reference)	1.48 (1.08, 2.03)	0.014
Adjusted odds ratio (95%CI), model 3	1.00 (Reference)	1.40 (1.02, 1.93)	0.037
Living alone (yes)			
MVPA, n	43	31	
Crude odds ratio (95% CI)	1.00 (Reference)	1.88 (0.97, 3.69)	0.062
Adjusted odds ratio (95%CI), model 1	1.00 (Reference)	1.68 (0.80, 3.58)	0.17
Adjusted odds ratio (95%CI), model 2	1.00 (Reference)	1.69 (0.77, 3.72)	0.18
Adjusted odds ratio (95%CI), model 3	1.00 (Reference)	1.60 (0.72, 3.55)	0.25

A logistic regression model was used to calculate the odds ratios. Model 1 was adjusted for age and sex. Model 2 was adjusted for the same covariates used in model 1 plus self-reported health, smoking, educational background, subjective economic status, and depression status. Model 3 was adjusted for the same covariates used in model 2 plus social participation. MVPA: moderate-to-vigorous physical activity; CI: confidence interval

## Discussion

This cross-sectional study showed that Internet use was associated with sufficient physical activity (measured by a validated questionnaire) among community-dwelling older adults in Japan. These associations were observed even after accounting for potentially confounding factors, such as age, sex, lifestyle, phycological factors, and socioeconomic factors. Additionally, Internet use was associated with MVPA only for participants who were not socially engaged and lived with family members. Therefore, our study highlighted that the association between the Internet and physical activity is different in socially isolated older adults.

Many epidemiological studies suggest the benefit of Internet use for the health of older populations [32]. Today, the importance of engaging older adults using the Internet is being increasingly recognized to overcome their physical functional disabilities associated with social limitations because of COVID-19 [19, 33]. However, limited epidemiological evidence is available on the link between Internet use and physical activity during the pandemic-related social restrictions. A recent epidemiological study involving community-dwelling Japanese older adults showed that ICT, including Internet use, was associated with the implementation of exercise during the COVID-19 pandemic [21]. The results of this previous study are consistent with our results. Moreover, previous studies suggested that Internet use is affected by one’s economic status [33]. Another previous study found that older adult men with lower subjective economic status were likely to be less physically active [4]. However, the current study found that after controlling for subjective economic status, Internet users engaged in MVPA. That is, although the subjective economic status is an indicator of variance in life, such as income, social integration, and environment [20], our results suggest that promoting Internet use may be associated with maintaining and encouraging the physical activity of all older adults during a pandemic.

Many previous studies suggested that social participation promotes regular physical activity in older people [12, 13]. However, as of 2022, some pandemic-related restrictions on social activities remain in place, and older adults’ social participation in community activities is still low [34]. Even before the spread of COVID-19, approximately 34% of older people in Japan did not participate in social activities [16], and little is known about effective efforts to promote physical activity among this population. In this study, Internet use was associated with adequate physical activity only among participants who did not participate in social activities. A previous study showed that lower social participation was associated with lower physical activity in older adults during the COVID-19 pandemic [5]. Moreover, a study found that older people with lower level of physical activity showed a greater increase in physical activity due to the use of ICT [35]. This is likely because the effect of Internet use on increased physical activity was more pronounced in participants who did not previously participate in social activities. Therefore, community-dwelling older adults, especially those who do not participate in social activities, may be encouraged to use the Internet to maintain and promote their physical activity.

Several prior studies have suggested that older people who live alone are less physically active than those who live with their families [36, 37]. A previous study among Japanese older adults revealed that living alone was significantly associated with prolonged sedentary time [38]. Similar differences in physical activity by living arrangements were reported during the COVID-19 pandemic. A cross-sectional study involving community-dwelling Japanese older adults during the initial stage of the pandemic showed that individuals who live alone and are socially inactive had decreased physical activity [3]. Such lack of physical activity among older people living alone has been attributed to the lack of interaction with family members and the inability to gain valuable information about their health from their surroundings [39]. However, little was known about whether the association between Internet use and physical activity differs between older individuals who live alone and those who live with family. In the present study, Internet use was associated with adequate physical activity for only participants living with family members and not those living alone independent of social participation. Living alone is an indicator of older people’s social restrictions [11]. Those living alone may face greater challenges with health behaviors other than physical activity and thus may need more social support, such as efforts to provide older adults who live alone with adequate instruction to promote physical activity during a pandemic.

In this study, 62.2% of the participants used the Internet to obtain information, including medical and health information. This result is similar to that of a previous study wherein more than half of the users perceived the Internet as an important source of health information [20]. During the pandemic, many public organizations published information on the Internet about exercise programs and physical activities to prevent a decline in physical functions among older people, and access to this information may have led to an increase in physical activity [40]. However, it is estimated that 37% of the world’s population does not have access to the Internet, with older adults being the most vulnerable [41]. According to a government survey of Japan’s adult population in 2020, among older adults, 82.7% of those aged 60–69 years, 59.6% of those aged 70–79 years, and 25.6% for those aged 80 years and older used the Internet, indicating a marked decline in Internet use as people age [42]. In addition to promoting physical activity, Internet use is shown to promote wellbeing [43], support active aging [13], and possibly support the maintenance of cognitive function [44]. Therefore, efforts to ensure that older adults use the Internet and provide them with adequate instruction on its use are critical. According to a survey by Japan’s Ministry of Internal Affairs and Communications, the reasons given by the elderly for not using the Internet are not knowing how to use it, worried about getting into trouble, and the high cost of using it [45]. Therefore, it is necessary for society, including private companies and local governments, to work toward supporting older adults’ digital use, especially those who have concerns about digital use. Specifically, this includes the creation of a system that enables people to learn and improve their digital literacy through online learning. It is also necessary to subsidize companies that conduct research and development of technologies related to communication and broadcasting services for older people [19].

The strengths of this study are the moderately large sample size and inclusion of people aged 65–90 years. In addition, we considered educational background and self-reported economic status. These are important determinants of Internet use, and the results remain significant even after adjusting for the confounding factors. Additionally, the response rate was acceptable (74.4%). However, this study also had several limitations. First, due to the cross-sectional design, we were unable to determine the causal nature of the association observed. Second, the measure of physical activity relied on a self-administered questionnaire, which could be susceptible to misclassification. Nevertheless, the questionnaire we used has been well-established, and many similar epidemiological studies have used it to assess physical activity [46, 47]. Finally, our study participants comprise residents of a local city and are not entirely reflective of community-dwelling older adults in Japan.

## Conclusions

Our data demonstrated the association between Internet use and sufficient physical activity, and this association differed depending on the social isolation of community-dwelling Japanese older adults. Future large-scale longitudinal studies are needed to determine the causal relationship between Internet use and an increase in physical activity, which may help provide public recommendations to promote physical activity among older people.

## Data Availability

All data generated or analyzed during this study are included in this article.

## Abbreviations

CI: Confidence interval
COVID-19: Coronavirus disease 2019
ICT: Information and communications technology
IPAQ-SF: International Physical Activity Questionnaire-Short Form
MET: Metabolic equivalent
MVPA: Moderate-to-vigorous physical activity
OR: Odds ratio
